# Comparison of clinical presentation and out-comes of Chikungunya and Dengue virus infections in patients with acute undifferentiated febrile illness from the Sindh region of Pakistan

**DOI:** 10.1371/journal.pntd.0008086

**Published:** 2020-03-23

**Authors:** Uzma Shahid, Joveria Q. Farooqi, Kelli L. Barr, S. Faisal Mahmood, Bushra Jamil, Kehkashan Imitaz, Zahida Azizullah, Faisal R. Malik, Dhani Prakoso, Maureen T. Long, Erum Khan

**Affiliations:** 1 Department of Pathology and Laboratory Medicine, Aga Khan University, Karachi, Pakistan; 2 Department of Biology, Baylor University, Waco, Texas, United States of America; 3 Department of Medicine, Aga Khan University, Karachi, Pakistan; 4 Department of Comparative, Diagnostic, and Population Medicine, University of Florida, College of Veterinary Medicine, Gainesville, Florida, United States of America; Fundacao Oswaldo Cruz, BRAZIL

## Abstract

**Background:**

Arboviruses are a cause of acute febrile illness and outbreaks worldwide. Recent outbreaks of Chikungunya virus (CHIKV) in dengue endemic areas have alarmed clinicians as unique clinical features differentiating CHIKV from Dengue virus (DENV) are limited. This has complicated diagnostic efforts especially in resource limited countries where lab testing is not easily available. Therefore, it is essential to analyse and compare clinical features of laboratory confirmed cases to assist clinicians in suspecting possible CHIKV infection at time of clinical presentation.

**Methodology:**

A prospective point prevalence study was conducted, with the hypothesis that not all patients presenting with clinical suspicion of dengue infections at local hospitals are suffering from dengue and that other arboviruses such as Chikungunya, West Nile viruses, Japanese Encephalitis virus and Zika virus are co-circulating in the Sindh region of Pakistan. Out-patients and hospitalized (in-patients) of selected district hospitals in different parts of Sindh province of Pakistan were recruited. Patients with presumptive dengue like illness (Syndromic diagnosis) by the treating physicians were enrolled between 2015 and 2017.

Current study is a subset of larger study mentioned above. Here-in we compared laboratory confirmed cases of CHIKV and DENV to assess clinical features and laboratory findings that may help differentiate CHIKV from DENV infection at the time of clinical presentation.

**Results:**

Ninety-eight (n = 98) cases tested positive for CHIKV, by IgM and PCR and these were selected for comparative analysis with DENV confirmed cases (n = 171). On multivariable analysis, presence of musculoskeletal [OR = 2.5 (95% CI:1.6–4.0)] and neurological symptoms [OR = 4.4 (95% CI:1.9–10.2)], and thrombocytosis [OR = 2.2 (95% CI:1.1–4.0)] were associated with CHIKV infection, while atypical lymphocytes [OR = 8.3 (95% CI:4.2–16.7)] and thrombocytopenia [OR = 8.1 (95% CI:1.7–38.8)] were associated with DENV cases at time of presentation. These findings may help clinicians in differentiating CHIKV from DENV infection.

**Conclusion:**

CHIKV is an important cause of illness amongst patients presenting with acute febrile illness in Sindh region of Pakistan. Arthralgia and encephalitis at time of presentation among patients with dengue-like illness should prompt suspicion of CHIKV infection, and laboratory confirmation must be sought.

## Author summary

Chikungunya and Dengue viral infection are prevalent in South Asia. These are spread by mosquito bites and present as acute febrile illness with clinical presentation difficult to differentiate. In this study we analysed clinical findings of patients with lab confirmed Dengue virus and Chikungunya virus infection and compared the clinical symptoms to assess significant features that can differentiate the two infections at time of presentation to clinics and hospitals. The study was conducted at the Aga Khan University Hospital, Karachi Pakistan. Our study confirms presence of Chikungunya virus as an etiologic agent of fever among patients with dengue like illness in Sindh province of Pakistan. Presence of joint pains, central nervous symptoms were more common in CHKV patients, while gastrointestinal symptoms and elevated liver enzymes were more commonly associated with dengue patients. Both viruses are endemic in Pakistan and clinical and lab diagnosis is essential.

## Introduction

Arbovirus infections are a disease spectrum associated with significant morbidity in humans. Amongst these, Dengue virus (DENV) and Chikungunya virus (CHIKV) share geographic and vector related features and mainly affect the tropics [1]. These vector-borne illnesses spread by the mosquitos of the *Aedes* family. While DENV belongs to the genus *Flavivirus*, CHIKV is a member of the genus *Alphavirus* in the family *Togaviridae*.

Both viral infections share similar disease manifestations including fever, rash and other non-specific findings. CHIKV presents after an incubation period of 2–3 days, as an abrupt onset of febrile illness with arthralgia and rash that resolves spontaneously in 7–10 days. However, in some patients, disease may progress to recurrent, debilitating polyarthralgia and poly-arthritis lasting for months to years [2]. Additionally, some patients may present with signs of central nervous system involvement, making it essential to diagnose.

First recorded in Tanzania during 1952–53, CHIKV has caused multiple outbreaks worldwide involving all continents but Antarctica [3]. CHIKV has been circulating actively in the Indian subcontinent for the last decade with reports of epidemic outbreaks from India to Sri Lanka. India reported around 1.4 million CHIKV infections in 2006 [4].

Despite this active transmission in the region, reports of CHIKV activity have been reported from Pakistan only very recently [3–5] with the first outbreak reported from Karachi in 2017 [6–9]. The true burden of this virus in the community is largely unknown especially in the backdrop of reports that 20–30% of clinically diagnosed DENV cases being negative for the virus; referred to in this study as non-DENV acute febrile illness (NDFI) [10–11]. Thus possibility of CHIKV infection misdiagnosed as dengue like illness.

In this study, we analysed subset of data of an active surveillance study for patients with acute febrile illness and/or suspected DENV infection in 5 cities of Sindh province in Pakistan. This study aimed firstly to ascertain the presence of CHIKV as etiologic agent of NDFI in Sindh province, and secondly, to compare clinical features of laboratory confirmed CHIKV cases with DENV infection to identify clinical features and laboratory findings that may help differentiate CHIKV from DENV infection at the time of clinical presentation.

## Methods

### Ethics statement

This study was approved by Institutional Review Board of the Aga Khan University Hospital (reference number 3183-PAT-ERC-14). Informed consent was obtained from all adult patients enrolled. The process of sample collection and questionnaire was explained to the children and adolescent patients in simple words. If they agreed, informed consent was taken from their parent(s)/ guardian(s).

A prospective, cross-sectional study was carried out to assess role of arboviruses namely: Chikungunya, West Nile and Japanese encephalitis virus as cause of fever in NDFI cases in the Sindh region of Pakistan from 2015–2017. Surveillance for febrile illness was conducted at five major cities: Karachi, Hyderabad, Sukkhar, Larkana and Mirpurkhas. The combined population of these cities is over 25.3 million and consists of a mix of urban, peri-urban and rural settings. For this comparative study, a subgroup analysis was performed on patients who tested positive for DENV and CHIKV.

### Patient selection and data collection

We recruited patients from 5 district hospitals one in each city. Patients presenting with acute febrile illness to out -patient department (OPD patients) and those admitted with dengue like illness (see details below) were recruited over a period of 3 years from 2015–2017. Appointed medical officers at each field site enrolled patients after taking written consent in accordance with the Declaration of Helsinki. In case of children less than 18 years, consent was sought from parents after the patients gave verbal assent to participation and sampling. Consent was taken in local language. Inclusion criteria was patients between 10–60 years of age with a fever >over 38oC (>100.4oF) for 3–10 days, with either of the following symptoms: headache, myalgia, arthralgia, vomiting, macular/ petechial rash, bleeding diathesis, altered mental status, or acute flaccid paralysis. Since the other causes of acute febrile illness had to be ruled out, i.e. typhoid by blood culture, malaria parasite by blood film and CBC to assist clinicians in patient management, in addition to serum collected for the viral studies. Consequently a large volume of blood sample (25 ml) was required, and this was the main reason for not including very young and very old patients. Thus, the exclusion criteria were age less than 10 years and >60 years, presence of chronic liver disease, haematological malignancies or chronic illnesses requiring use of steroids (>20mg/day of prednisolone for > 2 weeks, or other immunosuppressant). Non-probability consecutive sampling was used. Samples were de-identified and stored at -80°C after separation of serum. DENV and CHIKV testing was performed using a commercial IgM Enzyme-Linked Immunosorbent Assay (ELISA) (CHIKjj Detect IgM, InBios International, Inc, Seattle, WA) and reverse-transcription polymerase chain reaction (RT-PCR). Laboratory results, along with epidemiological and clinical data were recorded in a predefined questionnaire.

### Clinical and laboratory testing

Clinical diagnosis and patient management were performed according to the hospital’s standard of care. Details of the patient’s clinical and laboratory profiles were entered in the predefined data collection form by a trained research medical officer. Clinical diagnosis was defined as the provisional diagnosis made by the treating physician at the time of presentation before the etiological diagnosis was established by the laboratory tests.

**Fig 1:**
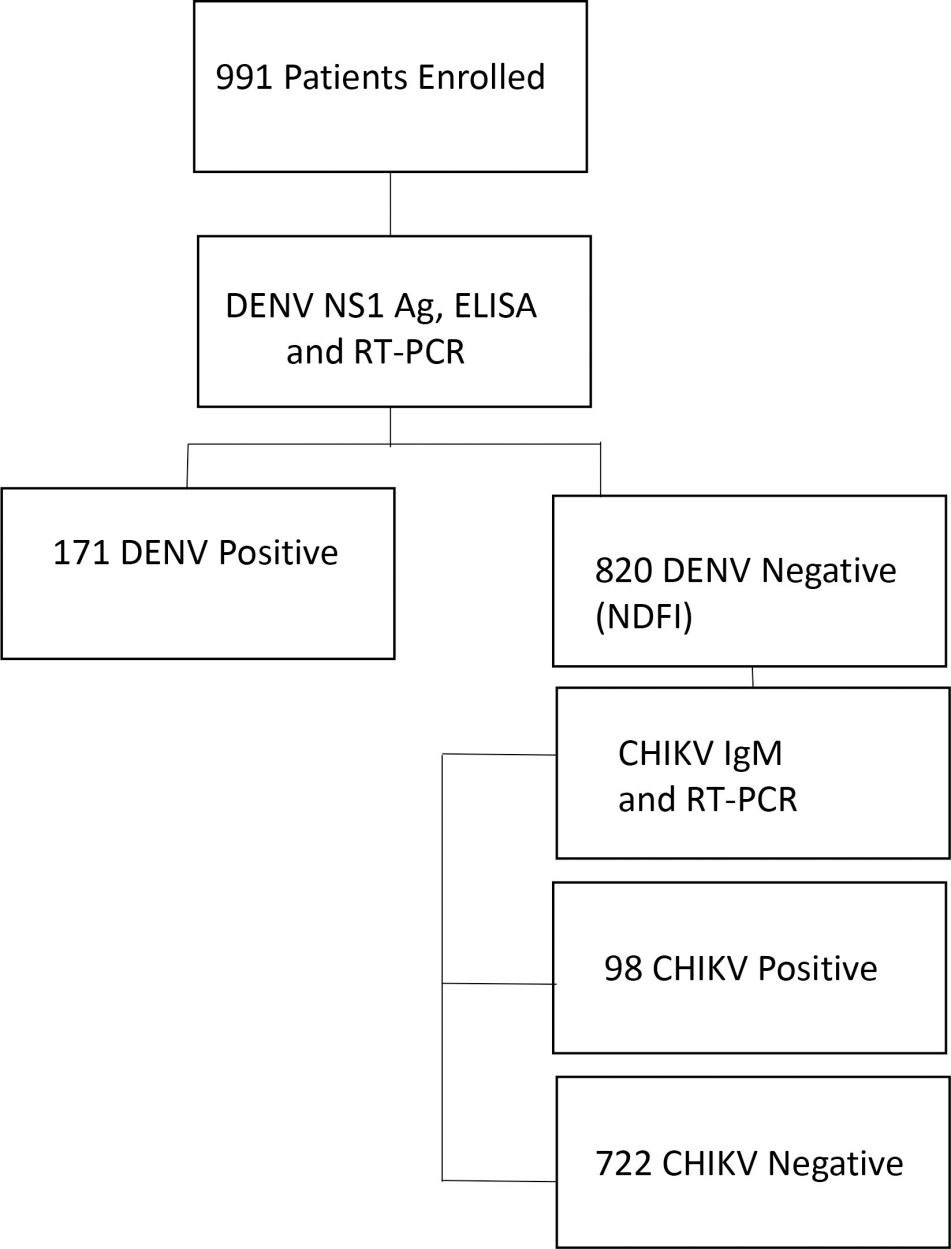
The diagnostic algorithm for DENV and CHIKV serologic testing. Flow chart illustrating patient recruitment according to inclusion criteria and workflow of specimen for DENV and CHIKV detection.

A laboratory diagnostic algorithm was followed (Fig 1). Samples that tested negative for DENV were considered as NDFI and were then evaluated for other viruses including CHIKV infection by IgM ELISA and RT-PCR. Hence, co-infection with dengue and chikungunya was not evaluated.

#### Serologic interpretation- ELISA

CHIKV and DENV immunoglobulin M (IgM) antibodies were detected via commercial IgM ELISA platforms (CHIKjj Detect IgM, InBios International, Inc, Seattle, WA), (DENV Detect IgM, InBios International, Inc, Seattle, WA) according to manufacturer instructions. These are sandwich-type immunoassays that detect human antibodies targeting the CHIKV and DENV envelope glycoproteins. Any indeterminate or borderline samples were retested and evaluated in duplicate to verify immune status. Dengue IgM was tested for cross-reactivity with other flaviviruses like West Nile, Japanese Encephalitis and Zika by Plaque Reduction Neutralization Test (PRNT). Dengue subtyping was done by PCR for antigen positive samples and PRNT for IgM positive samples.

#### RT-PCR

RNA extractions were performed using the QIAamp Viral RNA Mini kit (QIAGEN, Hilden, Germany) and RT-PCR performed as described elsewhere [8, 10]. A cycle threshold (Ct) value of <40 cycles was regarded as positive.

### Statistical analysis

Data from proforma was double entered into EpiData database. Data was then extracted in Microsoft Excel and transferred to SPSS 21.0 software for statistical analysis.

Frequencies and proportions were determined for qualitative variables, whereas, mean and standard deviation was calculated for quantitative variables. Pearson’s Chi-Square test and Independent t-test was used to identify associations between variables.

## Results

991 patients with a clinical diagnosis of DENV infection were enrolled. Of these 17.25% (n = 171) tested positive and confirmed as DENV infection. 11.95% (n = 98) of NDFI patients tested positive for CHIKV via IgM ELISA and RT-PCR. 79.6% (n = 78) cases of CHIKV were detected by IgM while 11.2% (n = 11/98) of samples tested positive for CHIKV by either ELISA or RT-PCR; fulfilling the CDC diagnostic criteria [12] for a confirmed infection. For Dengue fever, 63.1% (n = 108) of NS1Ag positive were also positive for IgM antibodies, while DENV was detected by PCR in 45.6% (n = 78) cases.

### Patient demographics

Patients with DENV were significantly younger than patients with CHIKV infection. DENV patients had a mean age of 33.6 years while CHIKV-infected patients had a mean age of 40.3 years (Table 1). More than 80% of patients in both groups were adults, with males comprising 56.7% and 55.10% of the population for DENV and CHIKV, respectively.

**Table 1:** Demographics of patients diagnosed with either DENV or CHIKV. Bold font indicates statistical significance.

	CHIKV	DENV	
Continuous variable	Mean ±SD	Mean ±SD	Difference (95%CI)
Mean age [years]	40.2 **±**17.1	33.6 **±**15.1	**6.6 (2.5 to 10.7)**
**Categorical variables**	**n = 98 (%)**	**n = 171 (%)**	**OR (95%CI)**
Children	10 (10.2)	22 (12.9)	0.8 (0.3–1.7)
Adults	88 (89.8)	149 (87.1)	1.2 (0.6–1.9)
Male	54 (55.1)	97 (56.7)	1.0 (0.8–1.2)
Female	44 (44.9)	74 (43.2)	1.1 (0.8–1.4)
**Location (total recruited)**			
Karachi	89 (90.8)	149 (87.1)	-
Mirpurkhas	1 (1.0)	7 (4.1)	-
Sukkhar	2(2.0)	2 (1.2)	-
Larkana	1(1.0)	2 (1.2)	-
Hyderabad	5 (5.1)	11(6.4)	-

Demographics of patients with dengue and Chikungunya virus infections. Qualitative variable is represented with frequency count and proportion [n = (%)] whereas for quantitative variables, mean and standard deviation [mean (SD)] is given. OR = Odds ratio, 95%CI = 95% confidence interval.

The majority of patients with DENV were from Karachi (87.1%, n = 149), followed by Hyderabad (6.4%, n = 11) (Table 1). Karachi was also the site for most of the CHIKV positive cases (91%, n = 89), then Hyderabad (5.05%, n = 5) (Table 1). Upper Sindh showed less activity for both DENV and CHIKV infection with only 4% of DENV patients from Mirpurkhas and 1% from each Sukkhar and Larkana. For CHIKV infection, 2% of patients were enrolled from Sukkhar and 1% each from Mirpurkhas and Larkana.

### Clinical findings

#### Symptoms

Most patients presented with a fever of less than 7 days with mean duration of 4.2 days in patients with DENV infection as compared to 5.4 days in patients with CHIKV infection.

Average fever of patients with CHIKV was 100.5°F and patients with DENV had an average temperature of 100.3°F. While there were differences in average systolic blood pressure, respirations and pulse, these variables were not statistically significant.

While Glasgow Coma Scale (GCS) did not differ significantly between the 2 groups, patients with confirmed CHIKV infection frequently presented with severe musculoskeletal pain and central nervous system (CNS) related symptoms (such as altered mentation, confusion and loss of consciousness): this difference was statistically significant with a p-value of 0.001. Gastrointestinal (GI) symptoms including nausea, abdominal pain and vomiting were reported in 53.8% of DENV patients (p<0.001) (Table 2). Haemorrhage was significantly associated with DENV virus infection with 8% of patients presenting with this symptom (p = 0.040) (Table 2). Rash was seen in 15.3% and 15.2% of CHIKV and DENV cases respectively and was not a significant predictor of disease (Table 2).

**Table 2:** Clinical presentation and laboratory findings of acute febrile patients with Dengue virus and Chikungunya virus infection from Sindh, Pakistan 2015–2017 (Bold font indicates statistical significance).

	CHIKV	DENV	Difference (95%CI)	OR CHIKV (95%CI)	OR DENV (95%CI)
**Clinical presentation**	**Mean ±SD**	**Mean ±SD**			
Mean days of fever	5.4±5.4	4.2±1.9	**1.2 (0.1 to 2.4)**	**-**	**-**
Mean temperature°C	100.5±2.0	100.2±1.9	0.2(-0.3 to 0.8)	**-**	**-**
Pulse (beats per minute)	97.2±16.2	95±19.7	2.3(-3.0 to 7.6)	**-**	**-**
Respirations (breaths per minute)	21.2±4.3	21±6.9	0.1(-1.6 to 1.9)	**-**	**-**
Mean systolic pressure	121±23.0	115.7±15.4	5.3 (-0.7 to 11.3)	**-**	**-**
Mean GCS	14.2±2.4	14.9±0.9	-0.7 (-1.2 to -0.2)	**-**	**-**
**Symptoms**	**n (%)**	**n (%)**			
Gastrointestinal	26 (26.5)	92 (53.8)	**-**	**0.5 (0.3 to 0.7)**	**2.0(1.4 to 3.0)**
Respiratory	2 (2.0)	2 (1.2)	**-**	1.7 (0.2 to 12.1)	0.6 (0.1 to 4.0)
Neurological	21(21.4)	12 (7.0)	**-**	**3.0 (1.6 to 5.9)**	**0.3(0.2 to 0.6)**
Haemorrhage	2 (2.0)	14 (8.1)	**-**	**0.2 (0.1 to 1.1)**	**4.0 (0.9 to 17.3)**
Musculoskeletal	61 (62.2)	51 (29.8)	**-**	**2.1 (1.6 to 2.7)**	**0.5(0.4 to 0.6)**
Ocular	9 (9.2)	11 (6.4)	**-**	1.4 (0.6 to 3.3)	0.7 (0.3 to 1.6)
Rash	15 (15.3)	26(15.2)	**-**	1.0 (0.6 to 1.8)	1.0 (0.5 to 1.8)
**Laboratory parameters**	**Mean ±SD**	**Mean ±SD**			
Aspartate aminotransferase (IU/L)	60.9±30.1	158.8±137.1	**-97.9 (-125.5 to—67.2)**	**-**	**-**
Alanine aminotransferase (IU/L)	55.1±53.3	113.4±115	**-58.2 (-85.4 to—31.2)**	**-**	**-**
Haemoglobin (g/dl)	12.1±2.5	13.6±2.0	**-1.5 (-2.1 to -0.9)**	**-**	**-**
Haematocrit (%)	37.2±6.6	40.5±5.6	**-3.4 (-5.0to-1.7**	**-**	**-**
Total leucocyte count (x10^9^/l)	8.6±4.7	4.2±2.3	**4.5 (3.4 to 5.4)**	**-**	**-**
Platelets (x10^9^/l)	254.7±150.1	110.8±89.8	**143.5 (109.8 to 177.1)**	**-**	**-**
Altered red blood cell morphology seen	36 (36.7)	35 (20.5)	**-**	**1.8 (1.2 to 2.7)**	**0.6 (0.4 to 0.8)**
Atypical lymphocytes seen	28 (28.6)	149(87.1)	**-**	**0.3 (0.2 to 0.5)**	**3.0 (2.2 to 4.2)**

Comparison of clinical presentation and laboratory profile amongst patients with dengue and Chikungunya virus infections. Qualitative variable is represented with frequency count and proportion [n = (%)] whereas for quantitative variables, mean and standard deviation [mean (SD)] is given. GCS = Glasgow Coma Scale, OR = Odds ratio, 95%CI = 95% confidence interval.

### Laboratory tests

#### Haematological tests

Average haematocrit and haemoglobin levels were within normal range for both DENV and CHIKV patients though 34.1% of DENV patients exhibited elevated haematocrit p <0.001 (as compared to 20% of CHIKV patients). For DENV, thrombocytopenia (defined as platelet count <150,000/mm3). was a significant predictor of disease with 74.1% of patients exhibiting this phenomenon (p <0.001) as compared to 18% of CHIKV patients; almost 150 platelets less than CHIKV (Table 2). While both DENV and CHIKV infected patients exhibited, on average, elevated leucocyte counts, 87% of DENV infected patients presented with atypical lymphocytes and were 3 times more likely to present with this sign than patients infected with CHIKV (Table 2). Altered red blood cell (RBC) morphology was also a significant finding and was reported almost twice as frequently in CHIKV infected patients than DENV infected patients, with 36.7% of CHIKV patients showing elevate levels of misshaped RBCs compared to only 20% of DENV patients (Table 2).

#### Liver function tests

Upon enrolment, the mean alanine aminotransferase (ALT) and mean aspartate aminotransferase (AST) levels of patients with CHIKV were within the normal range (55.13 IU/L and 60.90 IU/L, respectively) while DENV patients had significantly higher mean levels of ALT (113.4 IU/L) and AST (158.7 IU/L) (Table 2).

Patients with DENV were more likely to have elevated liver enzymes, 88.4% AST (OR 3.6, 95% CI: 1.2–10.7) and 58.3% ALT (OR 2.6, 95% CI: 1.2–5.6) than patients with CHIKV (68.1% AST and 35.1% ALT).

#### Multivariable analysis

On multivariable analysis (not shown in tables), presence of musculoskeletal [OR = 2.5 (95% CI:1.6–4.0)] and neurological symptoms [OR = 4.4 (95% CI:1.9–10.2)], and thrombocytosis [OR = 2.2 (95% CI:1.1–4.0)] were associated with CHIKV infection, while atypical lymphocytes [OR = 8.3 (95% CI:4.2–16.7)] and thrombocytopenia [OR = 8.1 (95% CI:1.7–38.8)] were associated with DENV cases at the time of presentation.

#### Clinical management

At presentation, 83.0% of DENV patients were clinically diagnosed with a viral infection, while only 50% of patients in the CHIKV group were diagnosed with a viral infection (Table 3). 10.2% of CHIKV patients were diagnosed with a bacterial infection and 12% as having a non-infectious aetiology by physicians (Table 3). Malaria was suspected in 10% of DENV infected patients and 6% of CHIKV patients (Table 3).

**Table 3:** Clinical diagnosis and management profile with outcomes of patients suffering from acute Chikungunya and Dengue enrolled from Sindh, Pakistan [2015–2017].

Provisional diagnosis	Chikungunya n = 98(%)	Dengue n = 171(%)	OR for CHIKV (95%CI)	OR for DENV (95%CI)
Viral infection	49 (50)	142 (83.0)	**0.6 (0.5 to 0.7)**	**1.7 (1.3 to 2.0)**
Bacterial infection	10 (10.2)	12 (7.0)	1.5 (0.7 to 3.2)	0.7 (.3 to 1.5)
Malaria	6 (6.1)	17 (9.9)	0.6 (0.3 to 1.5)	1.6 (0.7 to 4.0)
Suspected non-infectious aetiology	12 (12.2)	12 (7.0)	1.7 (0.8 to 3.7)	0.6 (0.3 to 1.2)
**Therapy**				
Antiviral agent	10 (1.02)	4 (2.3)	**4.3 (1.4 to 13.4)**	**0.2 (0.1 to 0.7)**
Systemic steroids	5 (5.1)	6(3.5)	1.5 (0.5 to 4.7)	0.7 (0.2 to 2.2)
Fluid replacement	50 (51.0)	131 (76.6)	**0.6 (0.5 to 0.8)**	**1.5 (1.2 to 1.8)**
Transfusions	1 (1.0)	4 (2.3)	0.4 (0.1 to 3.8)	2.3 (0.3 to 20.2)
Anti-malarial	1 (1.0)	3 (1.7)	0.6 (0.1 to 5.5)	1.7 (0.2 to 16.3)
Analgesics	59 (60.2)	106 (61.9)	1.0 (0.8 to 1.2)	1.0 (0.8 to 1.3)
Alive	92/92 (100)	170/170	-	-
			**Difference (95%CI)**	
Mean length of hospital stay (days)	3.1±5.2	3.5±2.6	**-**0.3 (-1.5 to 0.8)	

Clinical diagnosis and management profile amongst patients with dengue and Chikungunya virus infections. Qualitative variable is represented with frequency count and proportion [n = (%)] whereas for quantitative variables, mean and standard deviation [mean (SD)] is given. OR = Odds ratio, 95%CI = 95% confidence interval.

Despite have fewer viral diagnoses, CHIKV infected patients were 4 times more likely to receive antiviral therapy (primarily acyclovir) than DENV patients (Table 3). While not significant, use of steroid therapy was documented in 5.1% of CHIKV patients as compared to 3.5% of patients with DENV (Table 3). Fluid replacement therapy was required in 76.6% of DENV patients and 51% of CHIKV infected patients (Table 3). Up to 60% of patients required analgesics in both groups (Table 3).

## Discussion

In this study, we show that CHIKV is an important cause of NDFI cases in the Sindh province (12.1%) cases. These values are in contrast to those reported from other countries in the Asian region. Reller et al from Sri Lanka, in 2015 reported acute CHIKV infection in 3.5% of patients with acute febrile illness, 82.7% remained undiagnosed after complete laboratory workup [10]. Similarly, studies from India have reported CHIKV prevalence of 9.6–25% while 37% remained undiagnosed [6,11]. In our study most cases were from Karachi; one of the most densely populated cities in the world with a variety of social/economic factors contributing to uncontrolled urbanization and poor sanitation standards. This study also included samples from cluster outbreak of CHIKV infection during November 2016 that made the headline news in the country with 30,000 patients suspected of CHIKV infection [9].

Co-circulation of DENV and CHIKV in Karachi reflects a progressive deterioration of municipal infra-structure and efforts to control mosquitos [13]. More concerning is the identification of DENV and CHIKV in less densely populated peri-urban and rural areas, which lack of basic public health facilities, piped water and municipal infrastructure, making future epidemics imminent as seen in neighbouring India [14], unless preventive strategies are implemented in a timely manner.

Clinically, longer duration of fever, severe arthralgia and neurological manifestations were significantly associated with CHIKV. Twenty-one percent of CHIKV positive patients presented with CNS manifestations. This may have prompted the use of acyclovir in management of patients with CHIKV infection at time of admission, in lieu of possible herpes encephalitis. Similar findings of CNS involvement at time of presentation have been reported from India where altered mental status was seen in 56.67% of CHIKV patients requiring admission to intensive care units [15]. Literature from most of the CHIKV endemic region (i.e. Indian Ocean, South Asia, the Pacific islands, Southern Europe, the Caribbean, and South America) have reported neurological manifestation of CHIKV [16].

A study in La Reunion reported 24.1% of CHIKV cases presenting with abnormal neurological signs and symptoms [17]. And a recent study from Pakistan reported neurological symptoms in almost half of patients with confirmed CHIKV infections [8].

GI manifestations were most pronounced in patients with DENV infection, with 53.8% of DENV cases as compared to 26.2% of CHIKV, a finding that may help differentiate the two infections in clinical setting. The association of DENV with GI symptoms has consistently been reported from other endemic regions. Ramos et al in their retrospective review of 8559 patients reported GI symptoms in 67% of DENV patients [18]. In vitro studies have also shown DENV to have a predilection for GI tissue, especially liver [19]. This may explain the elevated AST and ALT levels in the DENV group and other reports from endemic regions [20]. Fernando et al recommend testing for liver function enzymes after 4–5 days of illness, as low levels of liver enzymes at the early stage of disease may underestimate ongoing liver damage [21]. Similarly, a study from Bangkok estimated up to a ten-fold increase of ALT/AST from baseline in hepatitis associated with severe DENV cases [22].

These findings are significant for Pakistan as chronic hepatitis B and C along with other gastrointestinal infections are endemic in the population. This data should alert physicians to include DENV in the differential diagnosis of patients presenting with fever, hepatitis and acute gastrointestinal symptoms.

Pronounced musculoskeletal symptoms including polyarthralgia was reported in 62.2% of CHIKV patients as compared to 29.8% in DENV patients. These symptoms may have resulted in misdiagnosis of CHIKV infection as non-infectious arthritis by clinicians at time of presentation and resultant use of steroids in these patients.

No significant difference was seen in the frequency of rash in both groups. The presence of comparatively lower systolic pressures in DEN-V and increased levels of haematocrit likely reflect plasma leakage resulting from increased vascular permeability and third spacing. [18]. Fluid replacement and supportive management was required more frequently in DEN-V patients, a finding in concordance with other reports from Pakistan [23, 24] and other parts of dengue endemic region [1,25].

Higher leucocyte counts and atypical lymphocytes were observed for DENV. Significant thrombocytopenia was also found in DENV patients. This corroborates the findings of Lee et al in their study on CHIKV versus DENV infections in adults [1]. While case fatality has been associated with both DENV and CHIKV, all patients in our study survived, [15, 26–29].

For laboratory diagnostics, the Dengue Early Rapid NS-1 test was the most effective for DENV detection as most cases of DENV infection were detected using this assay. Many of these patients were also positive by RT-PCR. Most NS-1 negative cases in our study were also negative by RT-PCR, validating the clinical utility of the NS-1 assay. While factors such as specimen shipping and delayed preservation could be possible reasons for the false negative RT-PCR cases in our study population, our findings agree with Ahmed et al who found that the overall performance of DENV RT-PCR to be no better than NS-1 antigen detection [30].

For the CHIKV serological diagnosis, 11% were positive with both IgM ELISA and RT-PCR, while 31.3% were detected only by RT-PCR, indicating that use of IgM alone in clinical settings as diagnostic assay may be insufficient and may result in false negative cases. This may happen in early phase of disease when antibodies are not detectable due to neutralization with viral antigen.

Nucleotide sequencing reports have shown associations of specific mutations at residue 226 of membrane fusion glycoprotein E1 (E1-A226V) region with disease severity and better virus adaptability [31]. These mutations have been associated with abrupt epidemic outbreaks of CHIKV. A study from La Reunion reported 34.3% of the total island population infected with viral containing the E1-A226V mutation [31]. Whole genome sequencing studies are currently underway to identify any mutations in CHIKV isolates yielded from Pakistan.

Most of our CHIKV patients were adult males which is in concordance with CHIKV epidemic reports from India and other regions [32, 33]. One sero-survey conducted in Calcutta reported the highest infection rate adults aged 50–55 years and virtually no sero-positivity in young children [34]. Further studies are required to explore this apparent age predilection in our population especially in very young children as this group was not included in this study.

Our study is limited by the nested design on a cross-sectional surveillance of febrile patients who presented to health centres. Since there was no follow-up, the outcomes and long-term sequelae of CHIKV infections could not be assessed. Disease severity data was also not collected; hence the subgroup of severe dengue cases could not be compared with CHIKV cases admitted to the hospital. Another shortcoming was the testing algorithm: as only DENV NS1 negative cases were tested for CHIKV, co-infections and specific manifestations in such cases could not be determined.

## Conclusion

Our study confirms that CHIKV is present in Sindh region of Pakistan and is an important cause of acute febrile illness. Patients with CHIKV infection are more likely to present with severe arthralgia and neurological symptoms. The recent CHIKV outbreak in Karachi highlights the possibility of Karachi having an epidemic potential in future, unless timely preventive interventions are ensured. While the sample size and the study design was insufficient to calculate the actual prevalence of disease in the community, our findings raise concerns for the possible circulation of a virulent CHIKV genotype known for its high rate of CNS involvement.

## Supporting information

S1 Checklist STROBE checklist. (DOC)

S1 Table Primer and probe sequences used for Chikungunya and dengue virus detection. (DOCX)
