# Supplementary material for: Comparison of clinical presentation and out-comes of Chikungunya and Dengue virus infections in patients with acute undifferentiated febrile illness from the Sindh region of Pakistan
Source: PLoS Negl Trop Dis. 2020 Mar 23;14(3):e0008086. doi: 10.1371/journal.pntd.0008086 (PMC7141694; doi:10.1371/journal.pntd.0008086)
Supplement: S1 Table — (DOCX) [file pntd.0008086.s002.docx]

| CHIKV virus | Forward  primer | 5' GAGCATAYGGTTACGCAGATAG 3' |
| --- | --- | --- |
|  | Reverse  primer | 5' TRCTGGTGAYACATGGTGGTTTC 3' |
|  | Probe | 5' FAM-ACGAGTMATCTGCGTAYTGGGACGYA – BHQ-1 3' |
| DENV virus -1 | Forward  primer | 5' CAAAAGGAAGTCGYGCWATA 3' |
|  | Reverse  primer | 5' CTGAGTGAATTCTCTCTRCTRAAC 3' |
|  | Probe | 5' FAM- CATGTGGYTGGGAGCRCGC – BHQ – 1 3' |
| DENV virus-2 | Forward  primer | 5' CAGGYTATGGCACYRTCACRAT 3' |
|  | Reverse  primer | 5' CCATYTGCAGCARCACSATCTC 3' |
|  | Probe | 5' FAM-CTCYCCRAGAACGGGCCTMGACTTCAA – BHQ 1 3' |
| DENV virus-3 | Forward  primer | 5' GGACTRGACACACGCACCCA 3' |
|  | Reverse  primer | 5' CATGTCTCTACCTTCTCGACTTGYCT 3' |
|  | Probe | 5' FAM- ACCTGGATGTCGGCTGAAGGAGCTTG – BHQ-1 3' |
| DENV virus-4 | Forward  primer | 5' TYRTYCTAATGATGCTRGTCG 3' |
|  | Reverse  primer | 5' TCCACCYGAGACTCCTTCCA 3' |
|  | Probe | 5' FAM- ATGCGTAGGAGTRGGRAACA – BHQ-1 3' |
